# Cognitive Ergonomics: A Review of Interventions for Outpatient Practice

**DOI:** 10.7759/cureus.44258

**Published:** 2023-08-28

**Authors:** Jennifer Li-Wang, Alexandra Townsley, Rajani Katta

**Affiliations:** 1 English, Katta Dermatology, Houston, USA; 2 Psychology, Rice University, Houston, USA; 3 Internal Medicine, Baylor College of Medicine, Houston, USA; 4 Dermatology, University of Texas Health Science Center at Houston, Houston, USA

**Keywords:** practice management, cognitive load theory (clt), human factors and ergonomics (hfe), outpatient practice, practice efficiency, cognitive ergonomics

## Abstract

Doctoring is difficult mental work, involving many cognitively demanding processes such as diagnosing, decision-making, parallel processing, communicating, and managing the emotions of others. According to cognitive load theory (CLT), working memory is a limited cognitive resource that can support a finite amount of cognitive load. While the intrinsic cognitive load is the innate load associated with a task, the extraneous load is generated by inefficiency or suboptimal work conditions. Causes of extraneous cognitive load in healthcare include inefficiency, distractions, interruptions, multitasking, stress, poor communication, conflict, and incivility. High levels of cognitive load are associated with impaired function and an increased risk of burnout among physicians. Cognitive ergonomics is the branch of human factors and ergonomics (HFE) focused on supporting the cognitive processes of individuals within a system. In health care, where the cognitive burden on physicians is high, cognitive ergonomics can establish practices and systems that decrease extraneous cognitive load and support pertinent cognitive processes. In this review, we present cognitive ergonomics as a useful framework for conceptualizing an oft-overlooked dimension of labor and apply theory to practice by summarizing evidence-based cognitive ergonomics interventions for outpatient care settings. Our proposed interventions are structured within four general recommendations: 1. minimize distractions, interruptions, and multitasking; 2. optimize the use of the electronic health record (EHR); 3. optimize the use of health information systems (HIS); and 4. support good communication and teamwork. Best practices in cognitive ergonomics can benefit patients, minimize practice inefficiency, and support physician career longevity.

## Introduction and background

Doctoring as cognitive labor

In clinical medicine, physician brain power is a valuable cognitive resource. Doctoring requires a tremendous amount of cognitive labor, involving diagnosing, decision-making, parallel processing, and communicating with patients, their families, and other members of the care team. A physician’s cognitive resources should be carefully managed to improve patient care and decrease the risk of burnout.

According to Michael Privatera, Professor of Psychiatry and Medical Director of the Medical Faculty and Clinician Wellness Program at the University of Rochester,

“A clinician’s brainpower is a limited, highly trained resource. It should be budgeted and optimally used just as you consider budgeting other resources in healthcare delivery” [1].

Cognitive ergonomics is focused on establishing practices and systems that allow physicians to use their cognitive resources effectively. Given the high rates of burnout in recent years, combined with the increasing administrative burden on physicians, it is especially timely to consider cognitive labor and its contribution to burnout [1]. Since cognitive labor is generally less recognized compared to more material forms of labor, we first must establish an understanding of cognitive labor that draws from existing thinking in cognitive load theory (CLT).

Cognitive load theory

With origins in educational psychology, CLT boasts wide applications in many disciplines including healthcare [2]. CLT is based on the assumption that working memory is a limited resource, and our brain thinks better when we minimize extraneous cognitive load (load that is redundant or unnecessary to perform the task). Any task performed by a healthcare worker inherently involves a degree of cognitive resources and effort. This is known as the *intrinsic cognitive load* of a task [2]. It is important to note that intrinsic cognitive load may differ from individual to individual depending on their experience and existing cognitive resources; Szulewski et al. describe intrinsic cognitive load as the “relative complexity of information specific to a task and person.” In comparison, extraneous cognitive load is a non-essential load generated by inefficiencies in the system. For example, accurately documenting the treatment plan in an electronic medical record (EMR) is crucial for patient care, therefore contributing to intrinsic cognitive load. However, a poorly designed EMR can make documentation unnecessarily burdensome for clinicians, increasing extraneous cognitive load [3]. A third type of load called *germane cognitive load* is the effort associated with learning and committing information to long-term memory.

A significant contributor to the cognitive load of healthcare workers comes from affective (emotional) labor. Clinicians routinely must regulate their own emotions and care for the emotions of others (patients, families, staff, peers). Traditional educational models group affective work into an extraneous cognitive load, but more recent paradigms view emotion as inseparable from medical practice and therefore part of intrinsic cognitive load [2]. Regardless of which type of load affective work falls into, the relationship between cognitive function and emotion is well-documented in the literature [4]. One study found that in simulations of neonatal care, negative comments made by a patient’s family had significant, deleterious impacts on multiple processes including diagnosing, performing interventions, communication, and teamwork [5]. In addition to the more obvious types of cognitive work like diagnosing and decision-making, the affective work doctors perform can significantly drain cognitive resources as well.

In education, CLT has been used to theorize the optimal learning environment for students. In health care, CLT can help design a more optimal working environment that minimizes cognitive load and maximizes cognitive performance. This is cognitive ergonomics which is defined as the discipline concerned with how people can think and operate more effectively in a system. CLT is useful in this discussion because it serves as a mechanism for understanding how good cognitive ergonomics work. Best practices in cognitive ergonomics should minimize unnecessary cognitive load.

Cognitive ergonomics

While CLT focuses on an individual within the system, human factors and ergonomics (HFE) focus on systems design. Since cognitive resources are critical to the practice of medicine, what are the ways we can sustainably use and allocate these resources? The field of cognitive ergonomics is focused on this question and is a discipline about the optimal use of cognitive resources within a system. The broader field of HFE is a scientific discipline that “applies theory, principles, data and methods to design in order to optimize human well-being and overall system performance” [6]. Domains within HFE include physical, cognitive, and organizational ergonomics. Cognitive ergonomics is the branch of HFE specifically concerned with cognitive processes (e.g. mental stress, decision-making, human-computer interaction, etc.).

According to CLT, working memory is the key to cognitive processing. When working memory is being occupied by extraneous loads such as in situations of high stress, emotion, or distraction, then cognitive processes suffer and operate sub-optimally. Best practices in cognitive ergonomics anticipate common sources of extraneous load and apply interventions to counteract them or protect cognitive resources. In this narrative review, we draw from the scientific literature to present interventions that promote good cognitive ergonomics in outpatient practice.

Significance

Cognitive ergonomics is an important field of study because it seeks to improve the well-being of both patients and healthcare providers. Previous research demonstrates that high cognitive load in health care providers is related to decreased patient care quality, due to an increased risk of medical errors, procedure failures, and poor decision-making [7,8]. Supporting the cognitive ergonomics of providers enables them to more effectively care for patients.

High cognitive load in health care providers is also associated with an increased risk of professional burnout. Burnout is a condition characterized by physical and emotional exhaustion, depersonalization, and a lack of a sense of personal accomplishment. According to a national survey of physicians in 2021, 44% of participants reported at least one symptom of burnout [9]. Burnout was positively associated with task load, which is commonly used as a reflection of cognitive load. Task load in this study was measured by the validated National Aeronautics and Space Administration Task Load Index (NASA-TLX). The study also found that task load varied between specialties, suggesting certain features of their specialties may be more or less conducive to good practices in cognitive ergonomics. Since physician cognitive resources are limited and vulnerable to burnout, cognitive ergonomics are important for building sustainable systems to preserve physician brain power.

To our knowledge, this is the first literature review to apply cognitive ergonomics to outpatient medical practice. 

There is no standardized set of cognitive ergonomics interventions that will improve every outpatient practice, as each clinic carries its unique needs, workflows, and staff. This review provides a theoretical framework for understanding cognitive labor in healthcare, evidence-based interventions from the literature, and concrete examples of how practices may implement our recommendations. Most importantly, our hope is that this review increases appreciation of cognitive work as a discrete, significant part of doctoring and fosters dialogue about what "good cognitive ergonomics" might look like within specific outpatient practice settings.

Methods

The aim of this narrative review is to present cognitive ergonomics interventions for outpatient practice. We conducted a literature review in PubMed of the following search terms: “cognitive ergonomics”, “cognitive load,” “cognitive task load,” and “cognitive interruptions and distractions” in health care. Cognitive load, workload, and task load are used interchangeably in this review. Only articles in English on improving the cognitive processes of healthcare providers working in care delivery were included in the review. Articles about information technology (e.g. computerized physician order entry (CPOE) and clinical decision-making systems) that did not present actionable interventions for healthcare providers were not included in this review.

Interventions for improving cognitive ergonomics were drawn from relevant articles. As most studies were conducted in inpatient settings, interventions were adapted to outpatient practice.

## Review

Evaluation metrics of cognitive load

Cognitive load can be measured by a variety of subjective and objective methods. On the subjective side, some studies rely on self-reporting by participants. NASA-TLX is a widely-used self-assessment to measure perceived cognitive load and is validated for use in health care workers [10]. NASA-TLX consists of a six-item inventory that asks takers to evaluate their mental demand, physical demand, temporal demand, performance, effort, and frustration when performing a task [11]. One review of the cognitive workload associated with electronic health records (EHR) found the most commonly used psychometric instrument was NASA-TLX [11].

Objective methods to measure cognitive load include pupil metrics: i.e. pupillometry (pupil size measurement), gaze, saccadic velocity (eye movement), and blink rate. Pupillometry relies on the principle that increased pupil size (dilation) signifies higher cognitive load, while decreased pupil size signifies lower cognitive load. High saccadic velocity (faster eye movements) occurs when cognitive load is low, while slower saccadic velocity occurs when the workload is high. Among these pupil metrics, the most reliable methods include pupillometry, gaze, and saccadic velocity, which have been validated in previous studies on some groups of healthcare practitioners including ultrasonographers and surgeons [12-14].

Observational studies are another objective method to measure cognitive load. In an observational study, a trained observer records and evaluates the behaviors of a subject using an established rubric. Observational studies may take place during simulations or *in vivo *clinical practice. Ten articles included in this review are observational studies.

In “Systematic review of measurement tools to assess surgeons' intraoperative cognitive workload,” Dias et al. recommended using multiple subjective and objective metrics to assess cognitive load [12]. In addition, given the dynamic nature of cognitive load, the article emphasized the importance of real-time tracking tools to give a more complete rendering of cognitive load over the course of a task or period of time.

Cognitive ergonomics interventions for outpatient practice

Recommendation A: Minimize Distractions, Interruptions, and Multitasking

Distractions and interruptions are defined as any events irrelevant to the task being performed and subsequently disrupt the clinic flow. Multitasking is the act of dividing attention between tasks, which consumes working memory and increases cognitive load.

Intervention #1: Identify which distractions, interruptions, or instances of multitasking are necessary for clinical practice.

Some distractions, interruptions, and multitasking are intrinsic to clinical practice. In a mixed methods study on critical care nurses, Potter et al. found that the cognitive load of registered nurses (RNs) was high not only due to the “task-related processes of patient care,” but also because of the many “cognitive shifts” nurses make when responding to the needs of different patients [8]. Similarly, physicians must divide their attention between multiple different patients and make timely judgments about their care. These cognitive shifts disrupt thinking but they constitute a necessary part of doctoring. Additionally, due to the dynamic nature of clinical medicine, some interruptions may be appropriate depending on the context (i.e. another care team member has a question concerning a patient’s care and must interrupt the physician from another task) [15].

Intervention #2: Identify which interruptions, distractions, or instances of multitasking are detrimental to clinical practice.

While some distractions, interruptions, and instances of multitasking may be necessary, they can still have detrimental cognitive effects. Distractions and interruptions make physicians vulnerable to making mistakes. In an observational study of emergency medicine physicians, physicians experienced an average of eight interruptions per hour [16]. Error rates increased significantly if physicians were interrupted or if they multitasked while prescribing.

Intervention #3: Identify and control the different sources of interruptions.

One study by Weigl et al. found that the most common physician workflow interruptions in hospitals were caused by telephones/beepers [17]. The cognitive load of devices should be weighed carefully against their utility, as many would consider phones as instrumental to their practice. In outpatient settings, physicians can consider when cell phone use is appropriate for their practice. For example, silencing phones during patient counseling or procedures may be considered.

Intervention #4: Identify times when interruptions and distractions will be best received.

Weigl et al. also found that the likelihood of certain interruptions happening depended on the tasks the physician was performing at the time [17]. For example, interruptions by colleagues and nurses were least likely when the physician was counseling a patient or in a meeting. Most interruptions occurred during charting and documentation activities.

Outpatient providers may consider designating times for outstanding non-urgent matters to be addressed without interrupting the clinic flow. Physicians should also dedicate time for charting without distraction.

Recommendation B: Optimize Use of the Electronic Health Record (EHR)

The EHR is a significant contributor to physician cognitive load. One eye-tracking study found that when documentation was less efficient, cognitive workload increased. The researchers reported that an “increase in the amount of time, number of keystrokes, and number of mouse clicks required to complete documentation increased cognitive workload” [18]. In other words, suboptimal EHR design can lead to increased cognitive load.

Intervention #5: Incorporate sufficient training for all EHR users.

A previous study showed cognitive workload increased for RNs during the transition from paper documentation to commercial EHR [19]. This increase was attributed to the cognitive effort of learning and navigating an unfamiliar EHR system. In another study surveying nurses, Heponiemi et al. found that sufficient implementation-related training decreased stress related to information systems (SRIS) among RNs while using the EHR [20].

The EHR has become a feature of modern medical practice, and previous studies demonstrate the importance of adequate EHR training for all staff to minimize excess cognitive load.

Intervention #6: Design EHR to prominently display the most important health data.

In an eye-tracking study of anesthesia personnel, visual attention to 15 areas of interest on the patient monitor was measured [21]. Results showed that a few vital signs commanded the most attention, namely blood pressure (BP), expiratory carbon dioxide, and electrocardiogram (ECG). As such, the authors recommended optimizing monitors to highlight the most relevant information.

In a simulation study of anesthesia staff, a novel “humanoid” avatar display format was used to show fictional patient monitor data during emergency simulations [22]. The study showed that the use of this novel display format corresponded to a 78% increased probability of correctly identifying the cause of an emergency as compared to the standard display. The authors also emphasized the importance of cognitive ergonomics in design, especially given the explosion of health data available to providers [22].

Most modern EHR systems allow some degree of customization to suit outpatient practice needs. Individual practices will value certain health data over others, and the EHR should be thoughtfully designed to minimize cognitive load while viewing pertinent data.

Intervention #7: Design and implement EHR changes that decrease cognitive load and improve patient care.

While the EHR introduces many challenges, it can meaningfully improve patient care. EHRs can incorporate built-in decision support for physicians that reduces cognitive load. For example, in one study, physicians were tasked with ranking five hypothetical patients by priority [23]. Physicians were presented with novel visualizations of simulated EHR data with decision support. The study concluded that well-designed EHRs that feature “clinically meaningful information patterns significantly reduced physician cognitive workload when prioritizing patient needs.”

Similarly, in a quality improvement (QI) study, residents using an enhanced usability EHR reported significantly lower cognitive workload and a 16% increased likelihood of appropriately managing test results, compared to residents using a baseline EHR [24].

Some EHR-based tools have emerged in recent years for a variety of specific health goals, such as the identification of pediatric hypertension and the prevention and management of childhood obesity [25,26]. Outpatient practices may be interested in developing or recruiting certain EMR-based tools for their practice.

Recommendation C: Optimize Health Information Systems

Health information systems (HIS) manage patient data using programs such as the EHR, practice management software, clinical decision support systems, and electronic prescribing systems. The following are interventions focused on optimizing HIS, not including the EHR.

Intervention #8: Utilize HIS to support-not supplant-the cognitive work of prescribing medications.

Some prescribing software performs automatic dosing according to standard practices. A review by Maslove et al. found that the use of computerized physician order entry (CPOE) reduced some medication errors but introduced new error types associated with the technology [27]. HIS should support best practices, but its use should not supplant the cognitive work required to determine the appropriate treatment for each unique patient case.

Intervention #9: Utilize HIS to support-not supplant-the cognitive work of other members of the care team.

According to a study by Beuscar-Zephir et al., CPOE is driven by a belief that “more exhaustive and more precise documentation efficiently prevents the risk of medication errors” [28]. In this observational study, the medication ordering process took place in two conditions: under synchronous cooperation (doctors and nurses round together) vs. asynchronous cooperation (the care team rounds separately). Under the synchronous condition, nurses actively participated in the medication ordering process and possessed a high level of understanding of the patient's case. In contrast, nurses in the asynchronous condition had limited decision-making, received limited communication, and operated at a “low level of process control.”

Some degree of automation supports good cognitive ergonomics by decreasing extraneous cognitive load for the physician and other members of the care team. However, it is important to remember that the cognitive labor of a skilled team is irreplaceable and the cognitive ergonomics of team members should be supported as well.

Recommendation D: Support Good Communication and Teamwork

Communication can transiently increase cognitive load, but in a team-driven field such as health care, good communication is crucial for good cognitive ergonomics and patient care. The value of teamwork also cannot be overstated, as high-quality teamwork has been shown to improve patient outcomes [29].

Intervention #10: Understand different kinds of communication failures.

In an ​​observational study of communication failures in the operating room, trained observers took field notes on content, audience, and purpose of communication exchange [30]. Communication failure was defined as a flaw in one of these dimensions.

Among 421 communication events noted, 129 constituted communication failures. Of these, 45.7% were "occasion" failures (poor timing), 35.7% were "content" failures (missing/inaccurate info), 24% were "purpose" failures (issues not resolved), and 20.9% were "audience" failures (key individuals excluded). Thirty-six percent of the communication failures resulted in "visible effects on system processes including inefficiency, team tension, resource waste, workaround, delay, patient inconvenience, and error” [30].

The physician and other members of the care team should be cognizant of common types of communication failures in order to improve teamwork.

Intervention #11: Design cognitive tools to support good communication.

In a study on emergency department handoffs, two cognitive tools were developed to assist physician communication during the shift change [31]. The tools were shown to improve communication, increase direct patient care time, and decrease time spent consulting medical records. The study recommended "in-depth observations and analyses of real work processes…to better support the quality of patient care” [31]. Similarly, a review of current communication practices among the outpatient care team would be valuable, particularly with an eye to developing communication tools to support the cognitive ergonomics of all team members.

A summary of the interventions presented in this review, with example applications in clinical practice, is noted in Table 1 below. We acknowledge these examples are by no means exhaustive or appropriate for every practice setting.

**Table 1:** Summary of cognitive ergonomics interventions with example applications for outpatient practice

Category	Intervention	Example Applications
Minimize Distractions, Interruptions, and Multitasking	Identify which distractions, interruptions, or instances of multitasking are necessary for clinical practice.	Conduct an audit of the frequency and nature of typical distractions or interruptions throughout the workday. With staff, establish what constitutes a necessary distraction or interruption and what can be addressed later.
	Identify which interruptions, distractions, or instances of multitasking are detrimental to clinical practice.	If an urgent task arises, switch attention completely. Allocate tasks to other staff to minimize multitasking.
	Identify and control the different sources of interruptions.	Conduct an audit of phone and email use throughout the day, and determine which may be delayed Triage interruptions and create workflows to capture tasks that can be addressed at a later time
	Identify times when interruptions and distractions will be best received.	Employ open vs. closed office door policies to control the timing of interruptions.
Optimize Use of the Electronic Health Record (EHR)	Incorporate sufficient training for all EHR users.	Target EHR fluency as a key goal for staff training. Establish where key information should be documented to reduce redundancy and search time among EHR users.
	Design EHR to prominently display the most important health data.	Choose an EHR designed with your specialty or customizability in mind. Identify customizable aspects of the EHR and make changes to suit practice-specific needs.
	Design and implement EHR changes that decrease cognitive load and improve patient care.	Explore EHR-based tools that add functionality relevant to the practice.
Optimize Health Information Systems	Utilize HIS to support—not supplant—the cognitive work of prescribing medications.	Ensure the accuracy of all prescriptions ordered via electronic services.
	Utilize HIS to support—not supplant—the cognitive work of other members of the care team.	Train staff to exercise good clinical judgment when ordering medications, lab work, etc.
Support Good Communication and Teamwork	Understand different kinds of communication failures.	Review with staff the communication events that are important for the practice. Discuss types of communication successes and failures that may occur.
	Design cognitive tools to support good communication.	Develop cognitive tools such as scripts or templates for pertinent communication events. Solicit feedback from staff about the communication they receive from the physician along with areas for improvement.

Limitations

There is limited literature on cognitive ergonomics in health care, let alone in outpatient settings. One limitation of this study is that most articles found in the literature search and included in this review focused on inpatient medicine. As such, the interventions may translate imperfectly into care settings outside the study context.

Another limitation is due to the variable definitions of cognitive load in the literature. Cognitive task load and cognitive workload were included as synonymous terms in this review, although conceptually these terms may be used in different ways. Some studies use definitions of cognitive load that may not completely capture what is being targeted with cognitive ergonomics. For example, multitasking draws heavily on working memory and increases cognitive load, which should be addressed by cognitive ergonomics. However, some studies may not explicitly include multitasking as a contributor to cognitive load.

Lastly, this study is limited in application due to the difficulty of measuring cognitive load in clinical practice. While multiple studies discussed different cognitive load metrics, few evaluated their feasibility for use. In outpatient settings, pupil metrics are less feasible due to associated costs and technology. Observational studies may be useful, but they require well-trained observers performing many hours of observation. In outpatient practice, self-assessments may be the most accessible and validated method of evaluating cognitive load.

## Conclusions

This review is the first to our knowledge to present cognitive ergonomics practices for outpatient practice. In health care, the cognitive load for physicians and other care team members is extraordinarily high. Cognitive ergonomics addresses this by exploring methods to decrease extraneous load and support pertinent cognitive processes. The implications of cognitive ergonomics in clinic practice include reducing physician burnout and improving patient care by reducing errors, redundancy, and inefficiency in the health system. Multiple measures can be used to measure cognitive load, with self-reporting (e.g. NASA-TLX) as the most accessible method for use in outpatient settings. The four main categories of cognitive ergonomics interventions for outpatient practice are: 1. minimize distractions, interruptions, and multitasking; 2. optimize the use of the EHR; 3. optimize the use of HIS; and 4. support good communication and teamwork. Further research is needed to explore cognitive load in outpatient settings specifically as well as to evaluate the effectiveness of cognitive ergonomics interventions in practice. Overall, cognitive ergonomics provides a novel framework to assess cognitive labor in health care, an invisible but crucial part of a doctor's workload. Supporting cognitive ergonomics in outpatient practice will improve the efficiency, quality, and longevity of clinical practice.
